# Anti-Inflammatory Effects of Fucoxanthinol in LPS-Induced RAW264.7 Cells through the NAAA-PEA Pathway

**DOI:** 10.3390/md18040222

**Published:** 2020-04-21

**Authors:** Wenhui Jin, Longhe Yang, Zhiwei Yi, Hua Fang, Weizhu Chen, Zhuan Hong, Yiping Zhang, Guangya Zhang, Long Li

**Affiliations:** 1 Department of Bioengineering and Biotechnology, Huaqiao University, Xiamen 361021, China; whjin@tio.org.cn (W.J.);; 2 Technology Innovation Center for Exploitation of Marine Biological Resources, Third Institute of Oceanography, Ministry of Natural Resources, Xiamen 361005, China; longheyang@tio.org.cn (L.Y.);; 3 Institute of Drug Discovery Technology, Ningbo University, Ningbo 315211, China

**Keywords:** fucoxanthinol, inflammation, NAAA, PEA, PPAR-α

## Abstract

Palmitoylethanolamide (PEA) is an endogenous lipid mediator with powerful anti-inflammatory and analgesic functions. PEA can be hydrolyzed by a lysosomal enzyme N-acylethanolamine acid amidase (NAAA), which is highly expressed in macrophages and other immune cells. The pharmacological inhibition of NAAA activity is a potential therapeutic strategy for inflammation-related diseases. Fucoxanthinol (FXOH) is a marine carotenoid from brown seaweeds with various beneficial effects. However, the anti-inflammatory effects and mechanism of action of FXOH in lipopolysaccharide (LPS)-stimulated macrophages remain unclear. This study aimed to explore the role of FXOH in the NAAA–PEA pathway and the anti-inflammatory effects based on this mechanism. In vitro results showed that FXOH can directly bind to the active site of NAAA protein and specifically inhibit the activity of NAAA enzyme. In an LPS-induced inflammatory model in macrophages, FXOH pretreatment significantly reversed the LPS-induced downregulation of PEA levels. FXOH also substantially attenuated the mRNA expression of inflammatory factors, including inducible nitric oxide synthase (iNOS), interleukin-6 (IL-6) and tumor necrosis factor-alpha (TNF-α), and markedly reduced the production of TNF-α, IL-6, IL-1β, and nitric oxide (NO). Moreover, the inhibitory effect of FXOH on NO induction was significantly abolished by the peroxisome proliferator-activated receptor α (PPAR-α) inhibitor GW6471. All these findings demonstrated that FXOH can prevent LPS-induced inflammation in macrophages, and its mechanisms may be associated with the regulation of the NAAA-PEA-PPAR-α pathway.

## 1. Introduction

Fatty acid ethanolamides (FAEs) are lipid mediators that exist ubiquitously in animal tissues. Anandamide (AEA), oleoylethanolamide (OEA), and palmitoylethanolamide (PEA) are the three primary FAEs that play key roles in the regulation of various physiological and pathological processes [1]. AEA and another endogenous lipid 2-arachidonoyl-sn-glycerol (2-AG) are the main members of endocannabinoids, which perform important roles in the treatment of obesity and metabolic disorders through cannabinoid receptors (CBRs) [2]. In comparison with AEA, OEA, and PEA have a lower affinity for CBRs, but primarily activate the nuclear receptor peroxisome proliferator to activate the receptor-α (PPAR-α), which have anorexic and anti-inflammatory effects [3]. FAEs can be deactivated through enzymatic hydrolysis to fatty acids and ethanolamide, thus limiting their bioactivities in the body. The major hydrolyzing enzyme for AEA is the fatty acid amide hydrolase (FAAH), which is extensively distributed in animal tissues and has hydrolysis effects on OEA and PEA [4]. Moreover, OEA and PEA can be degraded and inactivated by a lysosomal enzyme N-acylethanolamine acid amidase (NAAA), which expressed hydrolase at high levels in immune cells, especially macrophages [5]. The elimination of NAAA activity effectively controls inflammation by restoring endogenous PEA capacity via PPAR-α [6]. The administration of NAAA inhibitor is a good pharmacological strategy against inflammation and many inflammation-related diseases [7,8].

Fucoxanthin (FX) is an abundant marine carotenoid that is present in brown seaweeds, such as *Undaria pinnatifida, Hijikia fusiformis, Saccharina japonica*, and *Sargassum horneri* [9,10,11]. The beneficial health effects of FX include regulating obesity, diabetes, cancer, and inflammation [11,12,13,14]. Dietary FX is primarily hydrolyzed in the gastrointestinal tract by digestive enzymes to a deacetylated metabolite called fucoxanthinol (FXOH), which also plays essential roles in the regulation of obesity, diabetes, and cancer [15,16]. The anti-cancer effects of FXOH are more powerful than that of FX on the regulation of cell viability, apoptosis, and cell-cycle arrest [14,17]. Moreover, the anti-obesity effects of FXOH are stronger than that of FX [18].

Although the anti-inflammatory effects of FX have been extensively investigated in vitro and in vivo, only few studies have demonstrated the effects of FXOH in the modulation of inflammation [19,20,21,22]. FXOH can inhibit tumor necrosis factor-alpha (TNF-α) and monocyte chemoattractant protein-1 (MCP-1) mRNA expression in 3T3-L1 adipocyte cells co-cultured with RAW264.7 macrophage cells and suppress palmitic acid-induced inflammatory cytokine expression [23]. However, the directive effects of FXOH on lipopolysaccharide (LPS)-induced inflammation in macrophage and the corresponding mechanism remain unclear. In LPS-activated macrophages, NO and many pro-inflammatory cytokines were released as inflammatory mediators, leading to inflammation-related tissue injury [24]. As a target for anti-inflammatory agents, NAAA inhibition can effectively suppress the production of these inflammatory factors, depending on the activation of PPAR-α [25]. The present research aimed to investigate the effects of FXOH on the NAAA and FAAH activity and determine whether the NAAA/FAAH-FAE-PPAR-α pathway can mediate the beneficial effects of FXOH on LPS-induced inflammation in macrophage.

## 2. Results

### 2.1. Inhibitory Effects of FXOH on NAAA and FAAH Activity

**Figure 1:**
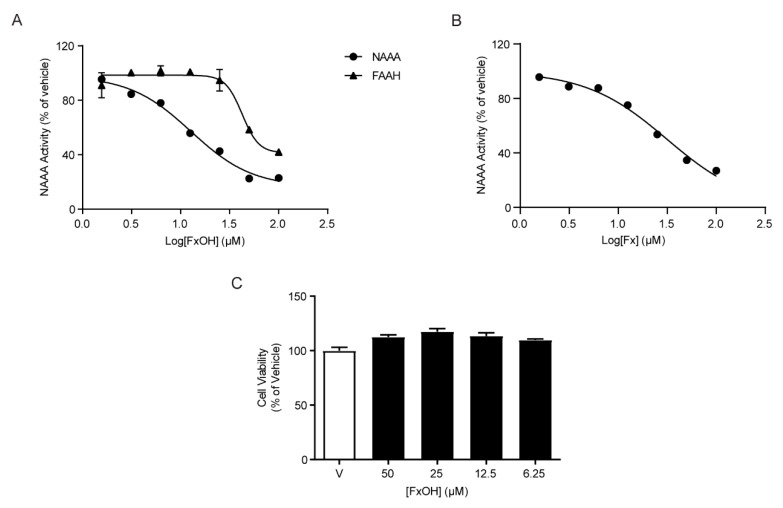
Inhibitory effects of fucoxanthinol (FXOH) on N-acylethanolamine acid amidase (NAAA) and fatty acid amide hydrolase (FAAH) activity. (**A**) Dose-dependent effects of FXOH on NAAA (filled circles) and FAAH activity (filled triangles). (**B**) Dose-dependent effects of FX on NAAA activity (filled circles). (**C**) Effects of FXOH on cell viability.

To first establish the inhibitory effects of FXOH on NAAA and FAAH activity, different concentrations of FXOH (1–100 µM) were incubated with recombinant human NAAA or FAAH protein. Results showed that FXOH exhibited much more powerful inhibitory activity for NAAA than FAAH (IC_50_ for human NAAA: 12.75 ± 1.12 µM, IC_50_ for human FAAH: 42.38 ± 1.11 µM, Figure 1A). To compare the difference of NAAA inhibitory activity between FX and FXOH, we also analyzed the inhibitory effect of FX on NAAA activity, and found that the NAAA inhibitory activity of FXOH was much powerful than that of FX (IC_50_ for human NAAA: 31.44 ± 1.06 µM, Figure 1B). To examine the potential toxicity of FXOH on RAW264.7 cells, we incubated the cells with 50, 25, 12.5, and 6.25 µM FXOH and assessed the cell viability. Results showed that FXOH has no significant effects on cell viability up to 50 µM (Figure 1C).

### 2.2. Molecular Docking Study of FXOH and NAAA

**Figure 2:**
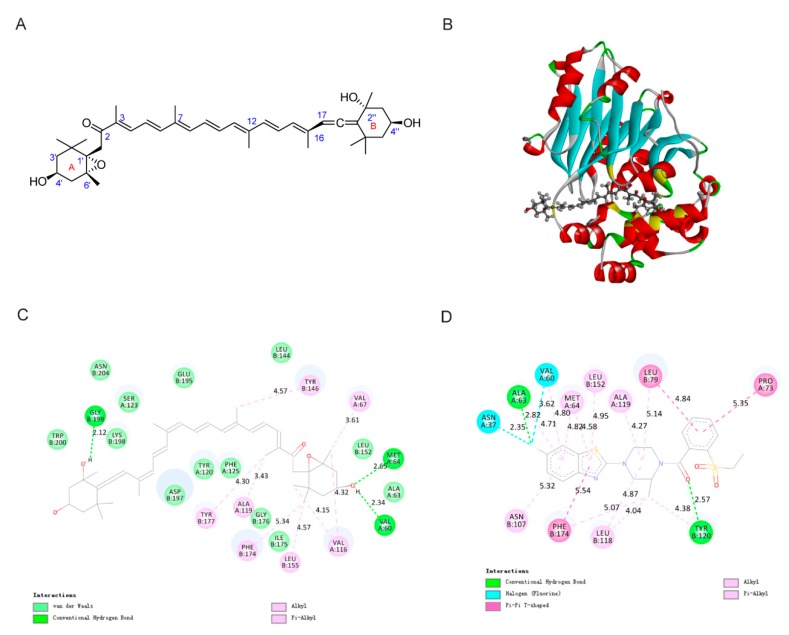
Interactions of FXOH with NAAA. (**A**) Chemical structure of FXOH; (**B**) interaction mode between FXOH and the active site of NAAA; (**C**) planar view of interactions between FXOH and NAAA; and (**D**) planar view of interactions between Arn19702 and NAAA.

The in vitro bioassay revealed the high inhibitory activity of FXOH on NAAA. To test whether FXOH interacted with NAAA and to predict their possible binding modes, we performed a molecular docking study for FXOH and NAAA (PDB code: 6DXX) by using Discovery studio 2019. The docking study revealed that FXOH was well docked into the pocket of the native ligand ARN19702 and shared multiple key active sites with Arn19702, such as ALA63, VAL60, MET64, ALA119, LEU152, and PHE174 (Figure 2C,D) [26]. As shown in Figure 2B, the A-side structure of FXOH was extended out of the ligand binding pocket (LBP), but the B-side structure was inserted deeply into the LBP of NAAA. Figure 2C shows the specific non-bonding interactions between FXOH and the residues of NAAA. The van der Waals, conventional hydrogen bond, alkyl, and pi-alkyl interactions were present between FXOH and NAAA. This finding indicates that hydrogen bonds and hydrophobic effects played a key role in the binding mode of FXOH and NAAA. Three hydrogen bonds interacted between FXOH and NAAA. The hydrogen atom of C4’’–OH formed a key hydrogen bond with B:GLY199 (distance: 2.12 Å), and the oxygen atom of C4’–OH formed two hydrogen bonds, which interacted with residues A:VAL60 (distance: 2.34 Å) and A:MET64 (distance: 2.65 Å). Additionally, hydrophobic bonds including alkyl and pi-alkyl interactions were observed between NAAA residues (A:VAL67, A:VAL116, A:ALA119, B:TYR146, B:LEU155, B:PHE174, and B:TYR177) and FXOH. Hence, FXOH can directly bind to the active site of NAAA.

### 2.3. FXOH Reversed LPS-Reduced Fatty Acid Ethanolamide Levels

**Figure 3:**
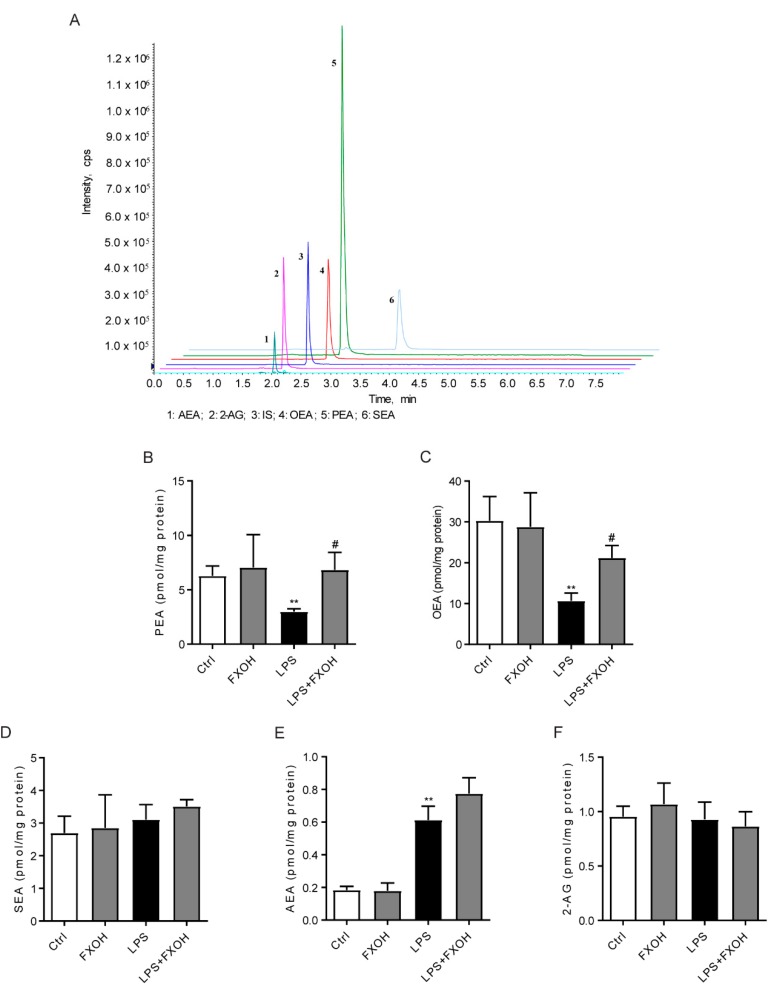
Effects of FXOH on fatty acid ethanolamide levels in LPS-activated macrophage. (**A**) LC–MS/MS chromatogram (**B**–**F**) palmitoylethanolamide (PEA), oleoylethanolamide (OEA), stearoylethanolamide (SEA), anandamide (AEA), and 2-AG levels were measured by LC–MS/MS assay. RAW264.7 cells were pre-treated with FXOH and then stimulated with lipopolysaccharide (LPS) for 24 h. Data represent the mean ± SEM. ** *P* < 0.01 compared with the control group, # *P* < 0.05 compared with the LPS + vehicle group.

Considering that the NAAA enzyme is highly expressed in macrophages and can hydrolyze FAEs to ethanolamine and fatty acid, LC-MS/MS was performed to further evaluate the effects of FXOH on NAAA activity and thus assess the effects of FXOH on FAE levels in LPS-stimulated macrophages (Figure 3A). Results showed that LPS significantly reduced the PEA and OEA levels (*P* < 0.01), and FXOH remarkably reversed the changes of PEA and OEA to a normal level (*P* < 0.05, Figure 3B,C). However, either LPS stimulation or FXOH treatment failed to modulate the levels of stearoylethanolamide (SEA) and 2-AG (Figure 3D,F). Interestingly, AEA significantly increased in response to LPS, which is consistent with previous findings [27,28]. Although no statistical difference was observed, FXOH seemed to enhance the increase of AEA induced by LPS, possibly due to the weak FAAH inhibitory effect of FXOH.

### 2.4. FXOH Suppressed the mRNA Expression of Inflammatory Factors

**Figure 4:**
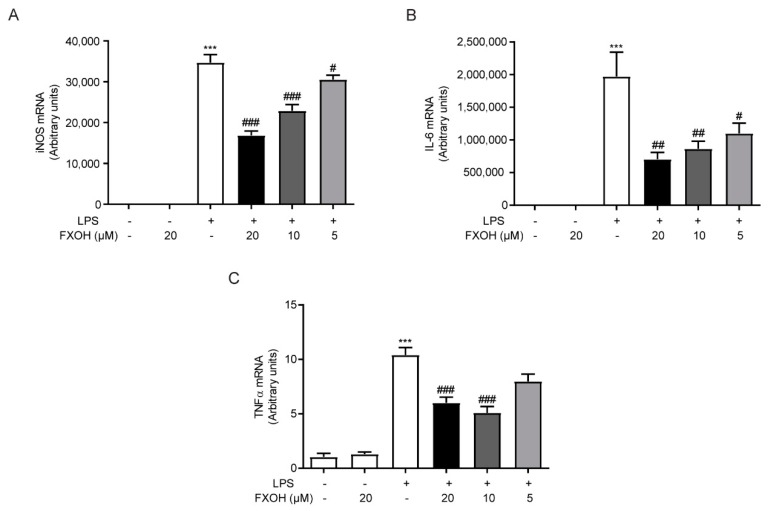
Effects of FXOH on the mRNA expression of inflammatory cytokines. (**A**) iNOS, (**B**) IL-6, and (**C**) TNF-α mRNA expression levels were determined by real-time PCR. RAW264.7 cells were pre-treated with FXOH (20, 10, and 5 µM) and then stimulated with LPS (100 ng/mL) for 24 h. Data represent the mean ± SEM. *** *P* < 0.001 compared with the control group, # *P* < 0.05 compared with the LPS group (received the treatment of LPS and the vehicle of FXOH), ## *P* < 0.01 compared with the LPS group, ### *P* < 0.001 compared with the LPS group.

To study the anti-inflammatory effect of FXOH in LPS-activated macrophages, we carried out real-time PCR to detect the mRNA expression level of inflammatory factors. Results showed that the mRNA levels of nitric oxide synthase (iNOS), interleukin (IL)-6, and TNF-α were significantly increased in macrophages induced by LPS (100 ng/mL) for 24 h (*P* < 0.001), while the increase of mRNA levels upon FXOH treated was significantly inhibited in a dose-dependent manner (Figure 4A–C).

### 2.5. FXOH Attenuated Cytokine Protein Levels and NO Production In Vitro

**Figure 5:**
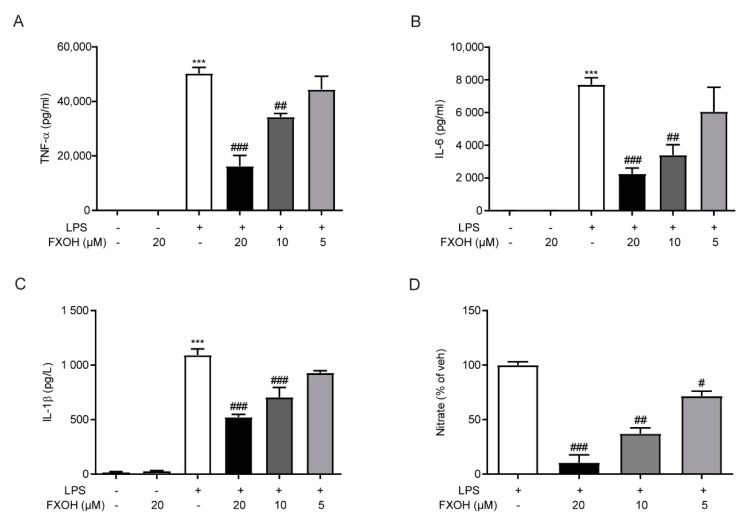
Effects of FXOH on cytokines protein levels and NO production in macrophage. (**A**) TNF-α, (**B**) IL-6, and (**C**) IL-1β protein levels were measured by ELISA; (**D**) nitrate expression in culture media was detected using a Griess Reagent kit. RAW264.7 cells were treated with 20, 10, and 5 µM FXOH 2 h prior to LPS (100 ng/ml) stimulation. Data are expressed as mean ± SEM. *** *P* < 0.001 compared with the control group, # *P* < 0.05 compared with the LPS group (received the treatment of LPS and the vehicle of FXOH), ## *P* < 0.01 compared with the LPS group, ### *P* < 0.001 compared with the LPS group.

When macrophages are activated by LPS, a large number of inflammatory cytokines are produced and secreted into the media of cell culture [29]. To evaluate the role of FXOH on protein levels of various inflammatory cytokines, we assessed the TNF-α, IL-6, and IL-1β levels in culture media by ELISA assay. Results showed that FXOH reversed LPS-induced cytokine expression in a dose-dependent manner (Figure 5A–C). Moreover, the production of NO is an important biomarker of inflammation. To evaluate the effect of FXOH on NO production, we detected the nitrate levels after FXOH treatment. Results showed that NO production in RAW264.7 cells was significantly inhibited in a dose-dependent manner (Figure 5D).

### 2.6. PPAR-α Mediated the Anti-Inflammatory Effects of FXOH

**Figure 6:**
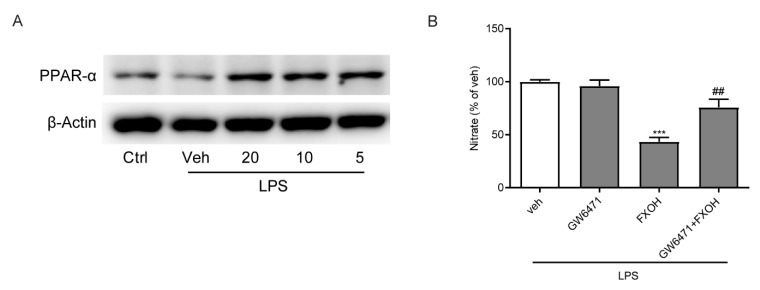
Effects of FXOH on LPS-induced macrophage activation were mediated by PPAR-α. (**A**) PPAR-α protein levels were measured by Western blot analysis and normalized against β-actin expression levels. (**B**) Nitrate concentrations in the culture media were evaluated using a Griess reagent kit. Data represent the mean ± SEM. *** *P* < 0.001 compared with the control group, ## *P* < 0.01 compared with the LPS + vehicle group.

The effects of NAAA inhibition on the regulation of inflammatory disorders depend on the PPAR-α signaling pathway [25]. Finally, to further determine the mechanism by which FXOH inhibits LPS-induced inflammation, we assessed the protein levels of PPAR-α through Western blot analysis. Results showed that LPS induced a marked decrease in PPAR-α expression, whereas FXOH dose-dependently reversed these changes (Figure 6A). Furthermore, GW6471, which is a specific antagonist of PPAR-α, was used to detect whether PPAR-α mediated the beneficial effects of FXOH in the control of NO production [30]. The results showed that GW6471 can significantly abolish the inhibitory effect of FXOH on LPS-induced nitrate expression (Figure 6B). Collectively, these data suggest that PPAR-α mediated the anti-inflammatory effects of FXOH in macrophages.

## 3. Discussion

In this present study, we proposed the inhibition of NAAA by FXOH as a new anti-inflammatory approach. Results indicated that FXOH significantly inhibited NAAA activity and increased PEA and OEA levels in the macrophage. Molecular docking assay demonstrated that FXOH was a potent NAAA inhibitor. As an NAAA inhibitor, FXOH can act as an anti-inflammatory agent in LPS-stimulated RAW264.7 macrophage cells. Considering that FXOH can markedly prevent LPS-induced mRNA expression levels of iNOS, IL-6, and TNF-α in the macrophage, it can also reduce NO production and decrease inflammatory cytokines in the culture media of macrophage, including TNF-α, IL-6, and IL-1β. Moreover, the inhibitory effects on nitrate expression were suppressed by the PPAR-α antagonist GW6471. The direct anti-inflammatory effects of FXOH in LPS-activated macrophage have not been reported. For the first time, we have indicated the ability of FXOH to suppress NAAA activity and restore PEA and OEA levels in the macrophage. Interestingly, although all kinds of NAAA inhibitors have been designed and studied in various diseases, we aimed to identify FXOH as the first NAAA inhibitor sourced from marine natural products.

PEA is a bioactive lipid with analgesic, anti-inflammatory, and neuroprotective effects in animals and in humans. Several studies have demonstrated the beneficial effects of PEA in murine experimental models. In a spinal cord injury model, the intraperitoneal administration of PEA markedly reduced tissue injury and inflammation via PPAR-α signaling [31]. In a mechanical hyperalgesia model, PEA significantly reduced hyperalgesia by inhibiting NF-kB signaling in mice [32]. In a rat model of stroke, PEA treatment reduced the neuronal apoptosis and inflammation in the brain through an intracellular mechanism [33]. The levels of PEA, a well-recognized anti-inflammatory lipid mediator, were significantly downregulated when stimulated by LPS in our present study, thereby supporting that PEA is downregulated following various inflammatory stimulation [34,35]. The inhibition of PEA degradation by targeting NAAA represents an alternative strategy for treating many diseases. Numerous NAAA inhibitors have been designed and regarded as efficient pharmacological tools in the control of various pathological conditions by reducing nicotine dependence [36], alleviating osteoarthritis development [37], and attenuating inflammatory and neuropathic pain [6].

Inflammation an immune response that occurs during pathogenic infection and various wound healing [38]. However, when the inflammatory reaction is excessive or when the body is in prolonged state of inflammation, it causes cell injury and tissue damage, thus causing or aggravating the development of a series of inflammation-related diseases, such as rheumatoid arthritis, asthma, cancer, ulcerative colitis, and Crohn’s disease [39]. Macrophage, the main type of immune cells in the body, is the major producer of inflammatory factors during inflammation-related diseases [40]. When exposed to danger signals such as LPS in microenvironment, macrophages can change its primary transcriptional program to increase the release of NO and induce the production of various inflammatory cytokines, including TNF-α, IL-6, and IL-1β [29,41]. The elevated expression of these mediators serves as a pathogenic agent in many inflammatory diseases, such as inflammatory bowel disease, asthma, and arthritis [42]. Herein, these inflammatory factors were markedly elevated in LPS-induced RAW264.7 cells, whereas the administration of FXOH significantly suppressed the expression of either mRNA or protein levels of these factors. These findings support the observations in previous studies by using NAAA inhibitors and provided good indication that FXOH may be a potential anti-inflammatory candidate drug. FXOH showed anti-inflammatory actions in many other inflammatory models. FXOH can attenuate TNF-α-induced MCP-1 and IL-6 expression and secretion in cultured adipocyte. FXOH can also significantly suppress palmitic acid-activated TNF-α, iNOS, and COX-2 expression in the macrophage [43]. However, few studies have fully demonstrated the anti-inflammatory mechanism of FXOH.

Three possible mechanisms can explain the anti-inflammatory effects of PEA. First, PEA can suppress the degranulation of mast cells [44]. Second, the ‘entourage effect’ occurs, where PEA may enhance the anti-inflammatory effects of AEA, which is often generated together with PEA, and activate CBRs [45]. Third, PEA can directly activate the nuclear receptor PPAR-α [46]. PPAR-α is a member of the highly conserved PPAR nuclear receptor superfamily, which was discovered in the 1990s. The PPAR superfamily has three subtypes, including PPAR-α, PPAR-β/δ, and PPAR-γ [47]. PPAR-α is the first identified member of this nuclear receptor superfamily, which is primarily distributed in metabolically active tissues, such as liver, kidney, heart. and skeletal muscle, and plays an important role in the regulation of lipid metabolism, glucose metabolism, and inflammation [48]. Therefore, PPAR-α agonists, which are primarily divided into synthetic fibrates and natural occurring agonists, are extensively used in the treatment of obesity, diabetes, and inflammatory diseases [49]. Natural ligands primarily include fatty acids and their derivatives, such as PEA and OEA, which can play metabolic regulation and anti-inflammatory effects through PPAR-α [34]. Our results showed that after the administration of FXOH, the levels of PEA were strongly upregulated, whereas the levels of AEA, SEA, and 2-AG in macrophages did not changed. Moreover, the PPAR-α antagonist, GW6471, can partly reverse the inhibitory effect of FXOH on NO production. Among the various inflammatory mediators released in the activated macrophages, NO is a sensitive messenger, which can be excessively produced in response to endotoxin stimulation, thus masking the development of inflammation [50]. Therefore, the NAAA-PEA-PPAR-α pathway is the main mechanism for the anti-inflammatory effects of FXOH in macrophage.

PPARα behaves as a potential therapeutic target for the treatment of inflammatory-associated disorders [51]. The mechanisms of how PPAR-α agonists exert anti-inflammatory activities have been well-studied primarily through the suppression of the transcriptional activity of NF-κB and AP-1 [52,53,54]. The autophagy pathways may also contribute to the PPAR-α induced suppression of inflammation [55]. The modulation of the NAAA-PEA-PPAR-α pathway is a rational therapeutic strategy for the control of inflammation in various inflammation-related diseases. The topical treatment with NAAA inhibitor ARN077 demonstrated a dose-dependent inhibitory effect on skin inflammation in DNFB-induced mouse allergic dermatitis [7]. Another NAAA inhibitor F215 showed anti-inflammatory effects both in LPS-induced acute lung injury model and TPA-induced local skin inflammation model [8]. The effects of FXOH in the present study on inflammatory control are comparable to those of PPAR-α agonists and other NAAA inhibitors. The present and previous findings suggest that FXOH regulates inflammation by increasing the levels of the endogenous PPAR-α agonists PEA and OEA. This conclusion is supported by the finding that the PPAR-α antagonist GW6471 abolished the effects of FXOH on nitrate expression control.

## 4. Materials and Methods

### 4.1. Chemicals and Reagents

GW6471 was obtained from MCE (Shanghai, China). Fucoxanthinol was prepared and identified by UV, MS, and NMR spectroscopy in our laboratory (purity ≥ 99%) [15]. LPS and all other chemicals were purchased from Sigma–Aldrich (Shanghai, China). All chemicals were dissolved in DMSO with various concentrations according to experimental need.

### 4.2. Cell culture and Treatment

We used the murine macrophage cell line RAW264.7, which was cultured by passaging every 2 days with Dulbecco modified eagle’s medium (DMEM) containing 10% fetal bovine serum (FBS). The culture was maintained in a cell incubator of 5% CO_2_ at 37 °C. To measure the fatty acid ethanolamide levels, we plated RAW264.7 cells in 10 cm dishes at a density of 4 × 10^6^ cells/dish and pre-incubated for 24 h, cells were pre-treated with FXOH for 2 h and then stimulated with LPS for 24 h. To examine the levels of inflammatory mediators, we seeded RAW264.7 cells into 12-well plates (3 × 10^5^ cells/well) at 24 h before treatment. The cells were then pre-treated with 20, 10, and 5 µM FXOH for 2 h followed by incubating with LPS (100 ng/mL) for 24 h. To determine whether FXOH on LPS-induced macrophage activation were mediated by PPAR-α, we pre-treated cells with GW6471 (10 µM) at 15 min before the treatment of FXOH and LPS. For all experiments, FXOH was dissolved in DMSO, LPS was dissolved in 1 × PBS (0.01M), and cells in the control group were treated with vehicle alone (DMSO or 1 × PBS).

### 4.3. CCK-8

RAW264.7 cells were inoculated in a 96-well plate. After pre-incubation overnight, 50, 25, 12.5, and 6.25 µM FXOH were added and incubated for 24 h. Then, the CCK-8 (MCE, Shanghai China) solution was added to each well of the plate, and the absorbance at 450 nm was detected by a microplate reader after 4 h of incubation.

### 4.4. NAAA and FAAH Activity Assay

The inhibitory roles of FXOH on enzymes were evaluated by enzyme activity determination as previously described [6]. Briefly, 30 μg of recombinant NAAA or FAAH proteins were cultured with FXOH or carrier (1% DMSO) at 37 °C for 10–30 min and with an analytical buffer containing 25 µM heptaenyl ethanolamide (Avanti lipses, Alabaster, AL, USA) or anandamide (Sigma Aldrich, Shanghai, China) as a substrate for NAAA or FAAH activity determination (NAAA pH 5.0, FAAH pH 8.0) for 10–30 min.

### 4.5. Molecular Docking Simulations

The molecular docking simulation of the compound FXOH was performed using Discovery Studio 2019 software (DS2019). The crystal structure of NAAA (human, PDB: 6dxx) was downloaded from the protein database (http://www.rcsb.org/pdb/explore/explore.do?structureId=6DXX). The structures of the ligands were generated using the DS2019 software, and the minimized energy was obtained from the CHARMm force field. Before docking, the NAAA crystal structure was cleaned, the protein was prepared, the missing ring region was simulated, the water was removed, the ligand was bound, and hydrogenation and other procedures were performed. The radius between the binding site and the original ligand is 10 Å. The docking program adopted part of the flexible program CDOCKER protocol. According to the energy fraction of CDOCKER, the interaction site, and the type of interaction with NAAA, the molecular docking results were evaluated [56].

### 4.6. RNA Isolation, cDNA Synthesis and Real-Time PCR

The total RNA from RAW264.7 cells was extracted using the TRIzol reagent (Thermo Fisher Scientific, Shanghai, China) following the manufacturer’s protocol. According to the manufacturer’s instructions, cDNA was synthesized with the main mixture of ReverTra Ace® qPCR RT Master Mix (Toyobo, Shanghai, China). Real-time PCR was performed on a Roche LightCycler 480 system by using SYBR® Premix Ex Taq™ II (Takara, Dalian, China), and the relative mRNA expression of each gene was normalized to GAPDH.

### 4.7. FAEs Extraction and Quantification

FAEs were extracted from Raw264.7 cells as previously reported [57]. Briefly, cells were harvested and ultrasonicated in 2 mL of methanol/water (1:1, v/v) containing 100 pmol of heptadecenoylethanolamide as internal standard. Lipids were extracted using 4 mL of chloroform, and the organic phases were collected and dried under N_2_. Lipids were resolved using 1 mL of chloroform and eluted in a silica column. The extract was eluted using methanol/chloroform (1:9, v/v) containing FAEs, dried under N_2_, and reconstituted in 100 μL of methanol for HPLC-MS/MS analysis. An AB 5500 Q-trap LC–MS/MS (ABSCIEX, Framingham, MA, USA) equipped with electrospray ionization and Agilent UPLC-1290system (Agilent Corp., Milford, MA, USA) was adopted in this experiment. The mass analysis and quantification of precursor/product ion transition in multiple reaction monitoring mode were carried out. The molecular ions were monitored at m/z 348.10/62.10 for AEA, m/z 379.10/287.30 for 2-AG, m/z 326.1/62.0 for OEA, m/z 300.1/62.1 for PEA, m/z 328.4/62.1 for SEA, and m/z 312.5/62.1 for C17:1 FAE as IS.

### 4.8. Cytokines and Nitrate Assay

The concentrations of mouse TNF-α, IL-6, and IL-1β in cell culture supernatants were analyzed using an ELISA kit (R&D Systems, Shanghai, China), and the quantitation of nitrite in cell culture supernatants was detected using a Griess reagent kit (Thermo Fisher Scientific, Shanghai, China).

### 4.9. Western Blot Assay

Western blot analysis was performed as previously reported [13]. The target proteins were detected using the primary antibodies mouse anti-PPAR-*α* (1:1000, Abcam, Shanghai, China) and mouse anti-*β*-actin (1:1000, Cell Signaling Technology, Shanghai, China).

### 4.10. Stasistical Analysis

All statistical calculations were performed using GraphPad Prism version 5.0 software. Data were expressed as mean ± SEM. Statistical analysis was completed by one-way analysis of variance (ANOVA) followed by Bonferroni’s multiple comparison test. *P* < 0.05 was considered to be statistically significant.

## 5. Conclusions

FXOH was identified as a new NAAA inhibitor to modulate PEA/ PPAR-α signaling in LPS-activated macrophages, thereby clarifying the mechanism of FXOH-induced anti-inflammatory effects and suggesting a new possible strategy against inflammatory diseases. However, future studies are needed to confirm the potential of FXOH by assessing its efficacy in various animal models of inflammatory diseases.
